# Management of Limited Interocclusal Distance with the Aid of a Modified Surgical Guide: A Clinical Report

**Published:** 2017-03

**Authors:** Farideh Geramipanah, Leyla Sadighpour, Amirreza Hendi

**Affiliations:** 1 Professor, Dental Research Center, Dentistry Research Institute, Department of Prosthodontics, Tehran University of Medical Sciences, School of Dentistry, Tehran, Iran; 2 Associate Professor, Department of Prosthodontics, Tehran University of Medical Sciences, School of Dentistry, Tehran, Iran; 3 Postgraduate Student, Department of Prosthodontics, Tehran University of Medical Sciences, School of Dentistry, Tehran, Iran

**Keywords:** Osteotomy, Stents, Dental Articulators, Denture, Complete

## Abstract

In completely edentulous patients, limited interarch distance can compromise conventional prosthetic fabrication. Bone reduction through various surgical procedures has been recommended to restore an acceptable interarch distance. In such circumstances, a surgical guide built on a mounted cast can be used to minimize and control the amount of bone reduction performed. In the present report, an innovative method of fabrication of surgical guide has been described.

## INTRODUCTION

Precise diagnosis and treatment planning are essential to achieve predictable outcomes and satisfactory results in patients, including those who are edentulous [1]. An organized protocol for obtaining a thorough diagnosis must be followed that includes taking dental and medical history, intra-oral and extra-oral examinations, making impression to produce diagnostic casts and mounting them on an appropriate articulator with the aid of jaw relation records [2].

Although the absence of teeth excludes several factors that could complicate diagnosis and treatment, other factors that remain relevant include the ability to provide retention and restore function while satisfying patients’ esthetic demands with a variety of denture base beds and maxillomandibular relationships. Accurately mounted casts are critical for the assessment of the prosthetic space [3]. The presence of exostoses and alveolar extrusion after tooth extraction may interfere with conventional recording of the jaw relationship and denture fabrication [4].

In severe circumstances, the exostoses and/or alveolar bone height must be reduced prior to prosthetic treatment; although such reduction must be kept to a minimum, as the bone is invaluable for denture support and retention [5–7]. Current trends endorse a selective stent-guided approach to site-specific bone recontouring, eliminating bony abnormalities that interfere with prosthetic reconstruction or insertion [8]. Hence, a guide template is critically important for estimation of the amount of reduction during a surgical session [9].

As in other procedures, use of a surgical guide may be necessary when a large amount of bone needs to be removed [10]. Several types of surgical guides are available, and the selection is based on the objectives and applications developed by the prosthetic clinician on the patient’s stone model. This clinical report describes a method for development of a specific template to guide precise amount of surgical bone reduction performed in an edentulous patient with limited interarch space.

## CASE REPORT

A 36-year old white female presented to the Department of Prosthodontics seeking a complete denture. She reported that she had become completely edentulous <1 year previously, and that all teeth had been lost due to excessive caries. No remarkable finding was identified in her medical record. Extraoral examination revealed no sign, symptom, or local condition that could have contributed to her maxillary jaw situation, including sinusitis and traumatic tooth extraction. Intraoral examination showed that the mucosal membrane was normal in terms of color, consistency and attachment to the underlying bone. The ridge had a hard bony consistency upon palpation, with no sign of displaceable or fibrous tissue. However, the entire maxillary residual ridge showed enlargement with severe prominences in the posterior region.

**Fig. 1:**
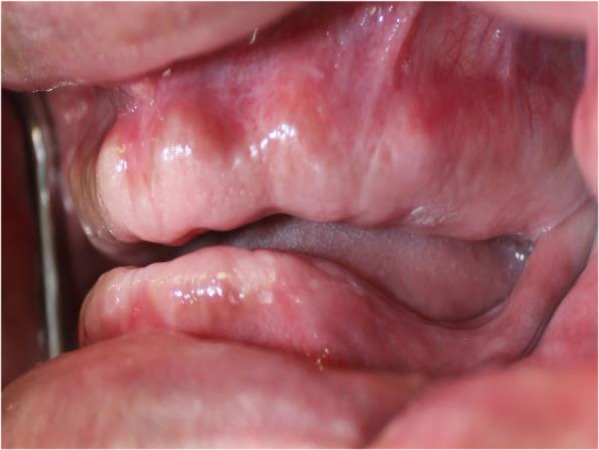
Amount of interarch space in rest position

**Fig. 2:**
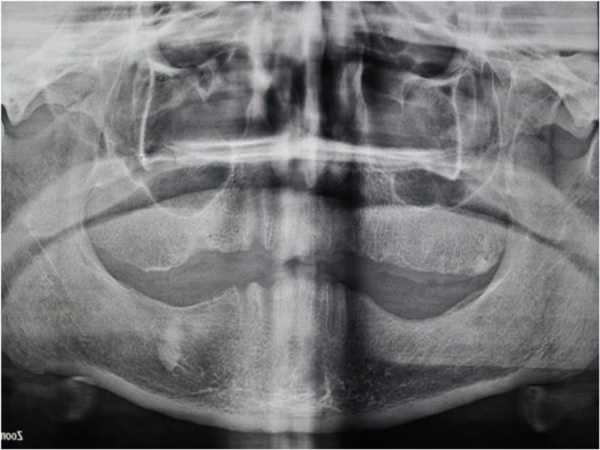
Panoramic view of patient reveals the amount of bone in maxillary tuberosity

**Fig. 3:**
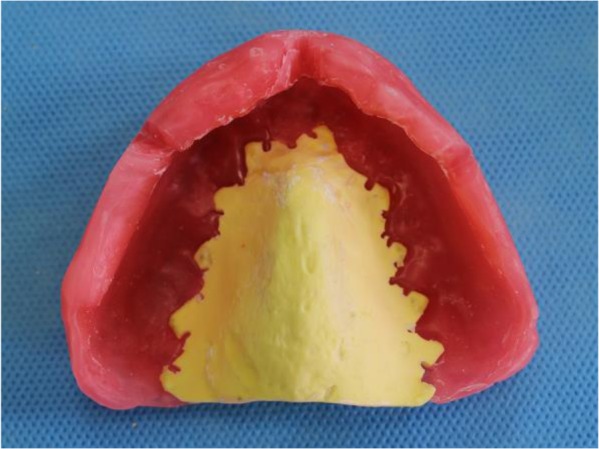
Wax-resin record base was made to overcome the limited space

In addition, maxillary tuberosity was very close to the mandible in the rest position (Fig. 1). A panoramic radiograph revealed maxillary ridge and tuberosity prominences with pneumatized sinuses (Fig. 2). Primary impressions were made using irreversible hydrocolloid material (Chromogel; Marlic Medical Industries Co., Tehran, Iran). The impressions were poured using type IV dental stone (GC Fuji Rock; GC Corporation, Tokyo, Japan). An acrylic resin record with wax rim bases could not be made for the maxillary arch due to extreme limitation of the interarch space. Thus, a combined acrylic-wax record base was fabricated to ensure sufficient strength and rigidity. The acrylic resin base was cut from all areas except the palate. Then, the modeling wax (Cavex, Haarlem, Netherlands) was adjusted along the residual ridge down to the vestibular areas (Fig. 3).

The mandibular record base was fabricated conventionally. After adjusting the anterior position of the maxillary occlusal plane with esthetic requirements, posterior position of the maxillary occlusal plane parallel to ala-tragus line, and mandibular occlusal plane with the height of lower lip in rest position, upper and lower lip support, and phonetics were examined. Subsequently, the vertical dimension of occlusion (VDO) was also established and moreover, a 3mm free-way space was verified between the VDO and mandibular rest position [11]. Finally, the centric relationship was registered and diagnostic casts were mounted on a non-Arcon semi-adjustable articulator (Hanau 96H2; Whip Mix Corporation, Louisville, KY, USA). On the mounted cast, the average height of mandibular wax rim was 5–6mm and maxillary wax rim was between 2–3mm and the average interarch space was 7mm (range 6–8mm), which was considered insufficient for complete denture insertion. Although implant supported prosthesis was offered as a treatment option, it was not accepted by the patient due to financial burden. Considering the limited free-way space of the patient (3mm), the option of VDO increasing was excluded. Hence, alveoloplasty was discussed with the patient, who consented to the procedure.

**Fig. 4:**
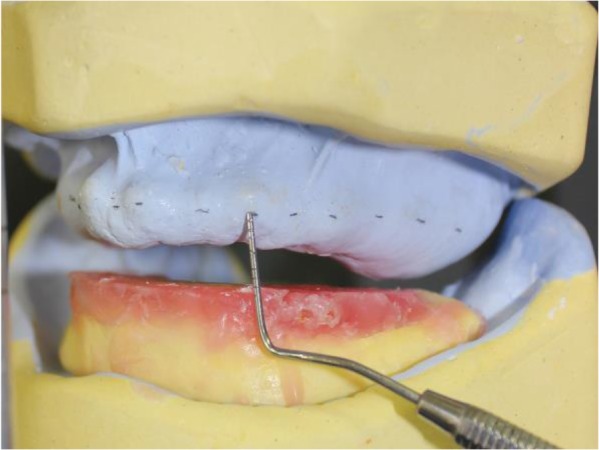
The amount of required space was marked on the working cast

**Fig. 5:**
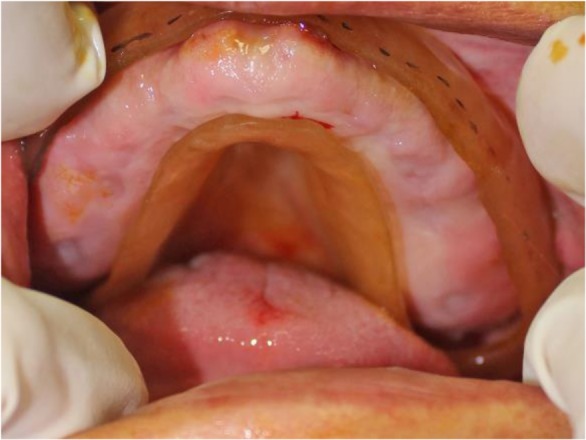
The first surgical guide in place

**Fig. 6:**
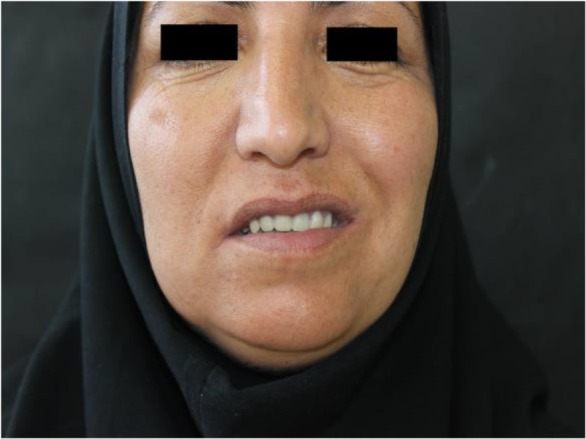
Patient with final prosthesis

According to the height of maxillary and mandibular wax rims, bone reduction of maxillary arch was inevitable. To avoid unnecessary bone reduction during surgery, a surgical guide was fabricated using the following procedure. With the mandibular wax rim in place on the mandibular cast, the total space required to set the artificial teeth plus a minimum 2-mm thickness for the acrylic resin base was calculated and marked on the maxillary arch (Fig. 4). Following the location of the line drawn by connecting the marks, a surgical stent was fabricated using auto-polymerizing acrylic resin (GC Corporation, Tokyo, Japan). A window was cut along the ridge, following the path drawn on the cast. No plaster was trimmed from the ridge to guide the surgeon to evaluate the amount of bone reshaping. An extra stent was made for the patient to wear in the first few postoperative days to maintain the surgical dressing. Alveoloplasty was performed under local anesthesia with the aid of the first surgical guide and a total of 2–4mm bone reduction was performed in the anterior and posterior regions of the maxilla (Fig. 5). After suturing, the second surgical stent was placed with surgical dressing. The healing phase was uneventful, and the patient returned six weeks postoperatively for the completion of treatment. As a result of alveoloplasty, she had gained 10mm interarch distance; thus, her treatment was completed following conventional procedures for edentulous patients (Fig. 6). She attended two post-delivery follow-up sessions in which minor adjustment was performed 24 and 36 hours after denture insertion. The patient was satisfied with the final results.

## DISCUSSION

The existence of bony prominences and undercuts in edentulous patients compromises the accurate establishment of the maxillomandibular relationship and appropriate arrangement of artificial teeth [4]. Although this situation could be addressed by increasing the VDO, this procedure did not meet the functional and esthetic demands of our patient. Moreover, increase in VDO may cause generalized patient discomfort, and/or neuromuscular symptoms [5,6,12]. In addition, metal palatal coverage was recommended in a similar situation [11]. However, in our patient, the remaining space was not even enough for retentive metal mesh and artificial teeth arrangement. The reduction of bone height has been recommended as an alternative [8]. Although alveoloplasty has the advantage of increasing the available restorative space without directly compromising the appropriate VDO, esthetics or phonetics, loss of cortical bone may be expected as an adverse outcome [12]. A detailed discussion of the surgical procedure for ridge correction falls beyond the scope of the present report. However, the value of bone preservation has been emphasized in the literature [2,6]. In the present case, a surgical guide was fabricated to aid the achievement of appropriate amount of bone reduction during alveoloplasty. Although prosthetic management techniques for patients with restricted interarch space have been reported, none of them was applicable to our patient due to the dentate status of those patients. For instance, Cheng et al, [3] reported that proper abutment selection enabled the placement of implant-supported prostheses in a patient with limited interarch space. Geckili et al, [5] described the treatment of a partially edentulous patient (Kennedy class II, modification I) with a limited interocclusal distance using orthodontic intrusion and massive alveolar resection. No information was provided regarding pre-prosthetic analysis of the amount of bone reduction required, and surgical guide fabrication was not mentioned. In the present case, the fabrication of a surgical guide and surgical stents imposed an extra expense on the patient. However, considering the importance of bone for the retention and support of a complete denture, this approach is justifiable in the long-term.
